# Long-term exposure to ambient air pollution and an increased risk of hyperuricemia

**DOI:** 10.1007/s10067-026-08398-z

**Published:** 2026-09-12

**Authors:** Su Hwan Cho, Hyun Jin Kim, Soontae Kim, Yoon-Hee Kang, Jae Moon Yun, Jin Ho Park

**Affiliations:** 1 Department of Family Medicine, Seoul National University Hospital, Seoul, Republic of Korea; 2 Department of Family Medicine, Seoul National University College of Medicine, Seoul, Republic of Korea; 3 National Cancer Control Institute, National Cancer Center, Goyang, Republic of Korea; 4 Department of Environmental and Safety Engineering, Ajou University, Suwon, Republic of Korea; 5 Environmental Institute, Ajou University, Suwon, Republic of Korea

**Keywords:** Air pollution, Hyperuricemia, Particulate matter, Uric acid

## Abstract

**Objectives:**

Evidence suggests that ambient air pollution contributes to metabolic dysregulation, including hyperuricemia. However, previous studies were limited to specific groups or regions with relatively high air pollutant concentrations. This study examined the association between long-term exposure to ambient air pollutants and hyperuricemia in a general population exposed to relatively low pollution concentrations.

**Method:**

This cross-sectional study analyzed 20,117 adults who underwent health screenings in the Republic of Korea (2015–2019). Annual mean concentrations of PM_2.5_, PM_10_, NO_2_, SO_2_, and CO were estimated at residential addresses using the Community Multiscale Air Quality model. Hyperuricemia was defined as serum uric acid level > 7.0 mg/dL (males) or > 6.0 mg/dL (females). Multivariable logistic regression estimated odds ratios (ORs) per interquartile range (IQR) increase in pollutant exposure, adjusting for confounders including estimated glomerular filtration rate.

**Results:**

Among 20,117 participants, 3,078 (15.3%) had hyperuricemia. All five pollutants were significantly associated with hyperuricemia. The ORs (95% CIs) per IQR increase were 1.06 (1.01–1.10) for PM_2.5_, 1.15 (1.08–1.21) for PM_10_, 1.07 (1.00–1.14) for NO_2_, 1.13 (1.07–1.19) for SO_2_, and 1.06 (1.01–1.11) for CO. Associations were stronger among current smokers, older adults, and individuals with dyslipidemia or central obesity (all *P* for interaction < 0.05).

**Conclusions:**

Long-term exposure to air pollutants was significantly associated with hyperuricemia, independent of renal function and even at low concentrations. These findings suggest that air pollution is an important environmental risk factor for metabolic disease, emphasizing the need for stricter regulations to protect vulnerable populations.

**Table:** 

Key Points • Long-term exposure to major ambient air pollutants is significantly associated with an increased risk of hyperuricemia, even at relatively low concentrations. • The association between air pollution and hyperuricemia persists independent of renal function, suggesting a potential role in systemic metabolic dysregulation. • Vulnerable populations, including older adults, current smokers, and individuals with metabolic conditions, exhibit stronger associations between air pollution exposure and hyperuricemia.

**Supplementary Information:**

The online version contains supplementary material available at https://doi.org/10.1007/s10067-026-08398-z.

## Introduction

Recent epidemiological studies indicated that the prevalence of gout and hyperuricemia has been rapidly increasing globally, imposing a substantial socioeconomic burden on healthcare systems [1]. Hyperuricemia was primarily considered a precursor to gout and kidney stones [2]. However, emerging epidemiological evidence has expanded its clinical significance, identifying it as an independent risk factor for various cardiovascular and metabolic diseases, including hypertension, atherosclerosis, chronic kidney disease (CKD), and type 2 diabetes [3–5]. Given its increasing prevalence and systemic health implications, identifying modifiable risk factors for hyperuricemia is important.

The etiology of hyperuricemia is multifactorial and involves genetic predispositions, dietary habits, and lifestyle factors. Ambient air pollution has recently attracted attention as a potential environmental determinant of metabolic dysregulation. Air pollutants, particularly particulate matter (PM_2.5_ and PM_10_), induce systemic inflammation and oxidative stress, which are key pathological pathways that may link environmental exposure to altered purine metabolism and elevated uric acid levels [6–9].

However, previous epidemiological studies examining this association have two notable limitations. First is regarding generalizability: most previous studies were restricted to specific occupational groups (e.g., traffic police officers or government employees) or narrow demographic segments (e.g., older adults), making it difficult to extrapolate the findings to the general population [10–13]. Second is regarding exposure intensity: the earlier studies were predominantly conducted in regions with high average air pollution concentrations. Consequently, the metabolic risks associated with exposure to relatively low concentrations, which are typical in many other developed nations, remain largely underexplored, creating a knowledge gap regarding potential low-level toxicity.

To address this gap, this study used a representative sample of the general population exposed to a wide range of air pollution levels, including relatively low concentrations. This study aimed to determine the independent association between long-term exposure to five major air pollutants (PM_2.5_, PM_10_, NO_2_, SO_2_, and CO) and serum uric acid levels or hyperuricemia after adjusting for potential confounders, including renal function.

## Methods

### Study population

**Fig. 1:**
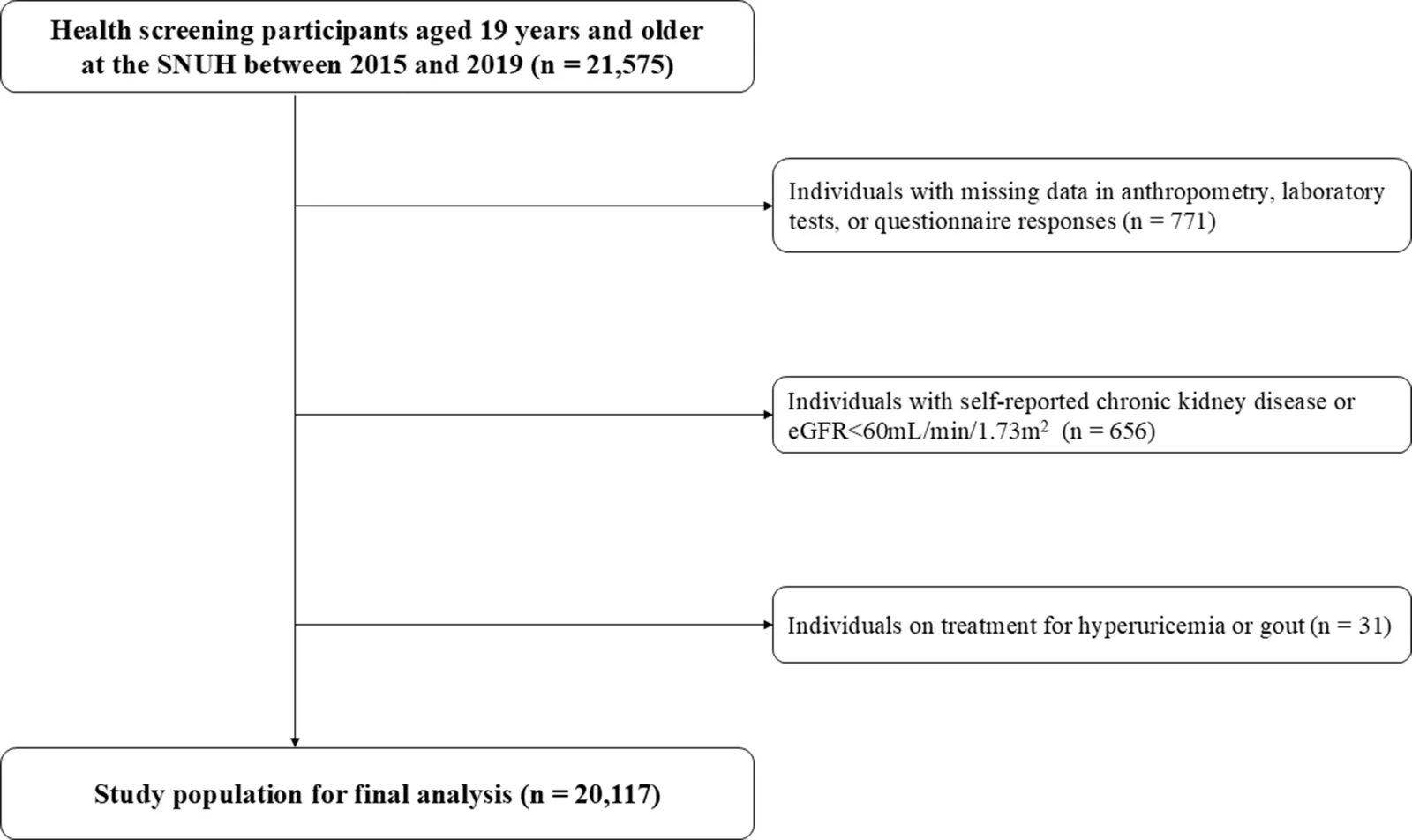
Flow chart illustrating the selection process of the study population

This study included adults aged ≥ 19 years who underwent a general health screening program at the Health Promotion Center of Seoul National University Hospital between 2015 and 2019. The screening program comprised anthropometric measurements, laboratory tests, major cancer screening, and surveys designed to obtain health-related information. Of the 21,575 participants, those with missing data on key variables were excluded. Additionally, participants were excluded if they had a self-reported history of CKD or an estimated glomerular filtration rate (eGFR) of < 60 mL/min/1.73 m^2^. The eGFR was calculated using the 2021 Chronic Kidney Disease Epidemiology Collaboration (CKD-EPI) creatinine equation [14]. Furthermore, participants receiving treatment for hyperuricemia or gout were excluded from the study. Consequently, 20,117 participants were included in the final analysis (Fig. 1). The study protocol was approved by the Institutional Review Board of Seoul National University Hospital (IRB No. H-2202–073–1299). The requirement for informed consent was waived owing to the retrospective nature of the study. All procedures were performed in accordance with the Declaration of Helsinki and its amendments.

### Air pollution exposure

Individual exposure to air pollutants, including particulate matter 2.5 (PM_2.5_), particulate matter 10 (PM_10_), sulfur dioxide (SO_2_), nitrogen dioxide (NO_2_), and carbon monoxide (CO), was estimated using a comprehensive modeling approach that integrated observational data with the Community Multiscale Air Quality (CMAQ) modeling system (version 4.7.1) [15]. To address the spatial limitations of ground-based monitoring stations (Air Korea, https://www.airkorea.or.kr/web), this study employed the CMAQ model with a 9-km grid resolution to estimate pollution levels across the entire country. This modeling system was coupled with the Weather Research and Forecasting (WRF, version 3.9) model for meteorological fields and the Sparse Matrix Operator Kernel Emissions (SMOKE, version 3.1) system for emission processing. The accuracy of the modeled concentrations was validated against ground measurements from the Air Korea Network, as detailed in previous studies [16, 17]. Daily average concentrations were aggregated for the 250 administrative districts and matched to each participant’s residential address. Regarding long-term exposure, each pollutant’s annual mean concentration was calculated for the year preceding the participant’s health-screening date (1-year lag).

### Definition of hyperuricemia and covariates

Hyperuricemia was defined as a serum uric acid level > 7.0 mg/dL in males and > 6.0 mg/dL in females, reflecting sex-specific differences in urate metabolism [18]. Information regarding medical history and health-related behaviors, including smoking, alcohol consumption, and physical activity, was obtained using self-administered questionnaires. Smoking status was categorized as “never smoked,” “previous smoker,” and “current smoker.” Alcohol consumption was classified as “non-drinking,” “light-to-moderate drinking,” and “heavy drinking.” Heavy drinking was defined as consuming ≥ 15 drinks/week or ≥ 5 drinks on a single occasion for males and ≥ 8 drinks/week or ≥ 4 drinks on a single occasion for females [19]. Adequate physical activity was defined as performing at least 150 min of moderate-intensity activity, 75 min of vigorous-intensity activity, or an equivalent combination thereof per week [20]. Comorbidities, including hypertension, diabetes mellitus, and dyslipidemia, were defined based on self-reported medication use or the following diagnostic criteria: systolic blood pressure ≥ 140 mmHg or diastolic blood pressure ≥ 90 mmHg for hypertension; fasting blood glucose ≥ 126 mg/dL or hemoglobin A1c ≥ 6.5% for diabetes; and low-density lipoprotein (LDL) cholesterol ≥ 130 mg/dL for dyslipidemia.

### Statistical analysis

Baseline characteristics were compared between participants with and without hyperuricemia using the Student’s t-test for continuous variables and the chi-square test for categorical variables. The data were presented as means (standard deviation [SD]) for continuous variables and numbers (percentages) for categorical variables. Pearson’s correlation analysis was performed to examine the association between air pollutants.

Multivariable linear regression analyses were performed to examine the association between air pollutant exposure (per IQR increase) and serum uric acid levels, treated as a continuous variable. Potential confounders were adjusted using three sequential models. Model 1 was adjusted for age and sex. Model 2 was adjusted for body mass index (BMI), waist circumference (WC), and lifestyle factors (smoking status, alcohol consumption, and physical activity). Model 3 was adjusted for eGFR and comorbidities, including hypertension, diabetes, and dyslipidemia. In these models, sex, smoking status, alcohol consumption, physical activity, and comorbidities were considered categorical variables, whereas age, WC, BMI, and eGFR were considered continuous variables.

Subsequently, multivariable logistic regression analyses were conducted to determine the ORs and 95% confidence intervals (CIs) for hyperuricemia associated with an IQR increase in air pollutant exposure, using the same adjustment models (Models 1, 2, and 3). The 1-year average exposure concentrations were used to assess the effects of long-term exposure.

To examine potential effect of modification by covariates, this study tested for interactions between air pollutants and key covariates in a fully adjusted model (Model 3). This was achieved by introducing a multiplicative interaction term into the regression model. Statistical significance of the interaction was assessed based on the interaction term’s *p*-value. Regarding continuous covariates (e.g., age and WC) that exhibited significant interactions, this study calculated and plotted the marginal effects of air pollutants across the range of the covariate. This visualization allowed interpretation of how the magnitude of the association changed as the covariate value increased. For categorical covariates (e.g., smoking status and comorbidities), stratified analyses were conducted to estimate the association within each subgroup. Heterogeneity of the effects between subgroups was evaluated using the interaction *p*-value. The results of these stratified analyses were visualized using a forest plot to facilitate comparison of effect sizes across subgroups.

All statistical analyses were performed using Stata software (MP, version 18.0; StataCorp, College Station, TX, USA) and R software (version 4.5.1; R Foundation for Statistical Computing, Vienna, Austria). Statistical significance was defined as a two-sided *p*-value of < 0.05.

## Results

### Study population’s baseline characteristics

Of the 20,117 participants, 3,078 (15.3%) had hyperuricemia. Compared with the non-hyperuricemia group, participants with hyperuricemia were younger and more likely to be male. The hyperuricemia group exhibited significantly less favorable metabolic profiles, characterized by higher BMI, WC, and blood pressure, as well as elevated levels of total cholesterol, triglycerides, and LDL cholesterol, along with lower HDL cholesterol. (*p* < 0.01). Regarding lifestyle factors and comorbidities, the hyperuricemia group exhibited higher rates of current smoking and heavy alcohol consumption and a greater prevalence of hypertension and dyslipidemia. Additionally, eGFR was significantly lower in the hyperuricemia group. Regarding air pollutant exposure, the hyperuricemia group had significantly higher annual average concentrations of all pollutants (Table 1).

**Table 1:** Study population’s baseline characteristics

	No HU	HU^†^	
	(*n* = 17,039)	(*n* = 3,078)	P-value
Age (years)	54.8 (12.9)	51.6 (13.1)	< 0.01
Sex (*n*, %)			< 0.01
Male	7,905 (46.4)	2,126 (69.1)	
Female	9,134 (53.6)	952 (30.9)	
BMI (kg/m^2^)	23.3 (3.3)	25.3 (3.8)	< 0.01
Waist circumference (cm)	83.4 (9.8)	89.4 (9.9)	< 0.01
Abdominal obesity (*n*, %)	1,419 (46.1)	1,659 (53.9)	< 0.01
Blood pressure (mmHg)			
Systolic	122.0 (17.3)	126.9 (16.6)	< 0.01
Diastolic	72.0 (11.2)	75.9 (11.4)	< 0.01
Uric acid (mg/dL)	5.0 (1.0)	7.6 (0.9)	< 0.01
Fasting glucose (mg/dL)	97.9 (22.3)	96.6 (21.0)	< 0.01
Hemoglobin A1c (%)	5.83 (0.72)	5.80 (0.60)	0.02
Total cholesterol (mg/dL)	196.0 (38.5)	208.2 (41.2)	< 0.01
Triglyceride (mg/dL)	107.3 (67.2)	144.6 (107.4)	< 0.01
HDL-cholesterol (mg/DL)	59.3 (16.3)	53.0 (15.5)	< 0.01
LDL-cholesterol (mg/DL)	118.8 (35.2)	130.7 (38.6)	< 0.01
eGFR^††^ (mL/min/1.73m^2^)	96.9 (13.1)	92.2 (14.6)	< 0.01
Smoking status (*n*, %)			< 0.01
Never	10,623 (62.3)	1,482 (48.2)	
Previous	3,863 (22.7)	860 (27.9)	
Current	2,553 (15.0)	736 (23.9)	
Alcohol drinking^†††^ (*n*, %)			< 0.01
No	8,114 (47.6)	1,090 (35.4)	
Light-to-moderate	4,508 (26.5)	693 (22.5)	
Heavy	4,417 (25.9)	1,295 (42.1)	
Physical activity^††††^ (*n*, %)			0.11
No	9,212 (54.1)	1,722 (56.0)	
Inadequate	1,526 (8.9)	278 (9.0)	
Adequate	6,301 (37.0)	1,078 (35.0)	
Comorbidities^†††††^ (*n*, %)			
Hypertension	5,192 (30.5)	1,206 (39.2)	< 0.01
Diabetes mellitus	2,243 (13.2)	356 (11.6)	0.02
Dyslipidemia	8,342 (49.0)	1,803 (58.6)	< 0.01
Concentrations of air pollutants^††††††^			
PM_2.5_ (μg/m^3^)	24.5 (2.5)	24.6 (2.5)	0.11
PM_10_ (μg/m^3^)	44.8 (5.2)	45.3 (5.3)	< 0.01
NO_2_ (ppb)	27.0 (8.8)	27.3 (9.0)	0.04
SO_2_ (ppb)	4.25 (0.90)	4.34 (0.93)	< 0.01
CO (ppb)	507.4 (70.5)	510.0 (72.3)	0.06

Data are presented as the mean (SD) and number (%)

^†^Hyperuricemia was defined as a serum uric acid level > 7.0 mg/dL in male and > 6.0 mg/dL in female

^††^eGFR was calculated using the CKD-EPI equation

^†††^Light-to-moderate alcohol consumption was defined as consuming < 15 standard drinks per week for male and < 8 standard drinks per week for female. Heavy drinking was defined as ≥ 15 standard drinks per week for male and ≥ 8 standard drinks per week for female, or consumption of ≥ 5 drinks for male and ≥ 4 drinks for female on a single occasion. (One standard drink = 14 g of alcohol)

^††††^Adequate physical activity was defined as at least 150 min per week of moderate-intensity or 75 min per week of vigorous-intensity aerobic physical activity, or an equivalent combination of moderate- and vigorous-intensity aerobic activity

^†††††^Hypertension was defined as taking antihypertensive medications or having a blood pressure of 140/90 mmHg or higher on the day of examination. Diabetes was defined as taking diabetes medications or having a fasting blood glucose ≥ 126 mg/dl or an HbA1c ≥ 6.5%. Dyslipidemia was defined as the condition where lipid-lowering medication was being taken or LDL-cholesterol was ≥ 130 mg/dl

^††††††^1-year average concentrations of air pollutants were defined as the concentration of the air pollutant that the participant was exposed to on an average for one year from the date of examination

*HU* hyperuricemia, *BMI* body mass index, *HDL* high-density lipoprotein, *LDL* low-density lipoprotein, *eGFR* estimated glomerular filtration rate, *PM* particulate matter, *NO*_*2*_ nitrogen dioxide, *SO*_*2*_ sulfur dioxide, *CO* carbon monoxide

### Air pollutants’ distribution and correlations

Table 2 presents descriptive statistics for the annual average concentrations of air pollutants to which participants were exposed. The mean concentrations (SD) of PM_2.5_, PM_10_, NO_2_, SO_2_, and CO were 24.55 (2.49) μg/m^3^, 44.90 (5.25) μg/m^3^, 27.04 (8.80) ppb, 4.26 (0.90) ppb, and 507.77 (70.78) ppb, respectively. The IQRs were 2.62 μg/m^3^ for PM_2.5_, 6.97 μg/m^3^ for PM_10_, 13.75 ppb for NO_2_, 1.10 ppb for SO_2_, and 83.71 ppb for CO. Supplementary Fig. 1 presents the Pearson correlation coefficients for the five air pollutants. Strong positive correlations were observed between PM_2.5_ and PM_10_ (r = 0.79) and between NO_2_ and CO (r = 0.85).

**Table 2:** Distribution of air pollutant exposure levels during the 1 year prior to the screening date

Air pollutants	Mean	SD	IQR	Percentile				
				10th	25th	50th	75th	90th
PM_2.5_ (μg/m^3^)	24.55	2.49	2.62	21.44	23.38	24.84	26.00	27.32
PM_10_ (μg/m^3^)	44.90	5.25	6.97	38.31	41.86	44.76	48.83	51.08
NO_2_ (ppb)	27.04	8.80	13.75	12.86	20.04	30.40	33.79	36.33
SO_2_ (ppb)	4.26	0.90	1.10	3.05	3.79	4.23	4.89	5.42
CO (ppb)	507.77	70.78	83.71	406.45	471.97	531.25	555.68	575.65

*SD* standard deviation, *IQR* interquartile range, *PM* particulate matter

### Air pollutants’ association with serum uric acid levels and hyperuricemia

The multivariable linear regression analysis (Table 3) indicated that serum uric acid levels were positively associated with increased air pollutant concentrations. In the crude and minimally adjusted model (Model 1), significant positive associations were observed only for PM_10_ and SO_2_. However, after adjustment for potential confounders, including lifestyle factors and comorbidities (Model 3), the associations between PM2.5 and NO2 became statistically significant. Consequently, in the fully adjusted model, an IQR increase in PM_2.5_ (*β* = 0.025, *p* = 0.002), PM_10_ (*β* = 0.063, *p* < 0.001), NO_2_ (*β* = 0.024, *p* = 0.047), and SO_2_ (*β* = 0.056, *p* < 0.001) was significantly associated with elevated serum uric acid levels.

**Table 3:** Association of 1-year exposure to air pollutants with serum uric acid levels

	Crude		Model 1^*^		Model 2^†^		Model 3^‡^	
	*β* (SE)	*p* value	*β* (SE)	*p* value	*β* (SE)	*p* value	*β* (SE)	*p* value
PM_2.5_	0.010 (0.010)	0.325	0.015 (0.009)	0.088	0.025 (0.008)	0.003	0.025 (0.008)	0.002
PM_10_	0.046 (0.013)	< 0.001	0.056 (0.011)	< 0.001	0.072 (0.011)	< 0.001	0.063 (0.010)	< 0.01
NO_2_	0.006 (0.015)	0.696	0.022 (0.013)	0.093	0.044 (0.013)	0.001	0.024 (0.012)	0.047
SO_2_	0.044 (0.012)	< 0.001	0.057 (0.010)	< 0.001	0.071 (0.010)	< 0.001	0.056 (0.009)	< 0.001
CO	0.001 (0.011)	0.923	0.011 (0.010)	0.250	0.026 (0.010)	0.006	0.017 (0.009)	0.067

The *β*-coefficients represent the change in serum uric acid levels for an IQR increase in the concentration of each air pollutant. (2.62 μg/m^3^ for PM_2.5_, 6.97 μg/m^3^ for PM_10_, 13.75 ppb for NO_2_, 1.10 ppb for SO_2_, 83.71 ppb for CO.)

^*^Adjusted for age and sex

^†^Adjusted for variables in model 1 and BMI, WC, smoking, alcohol consumption, and physical activity

^‡^Adjusted for variables in model 2 and eGFR, hypertension, diabetes mellitus, and dyslipidemia

*SE* standard error, *IQR* interquartile range, *PM* particulate matter, *BMI* body mass index, *WC* waist circumference, *eGFR* estimated glomerular filtration rate

Table 4 presents the ORs for hyperuricemia associated with an IQR increase in 1-year air pollutant concentrations. In the fully adjusted model (Model 3), significant positive associations were observed for all five air pollutants. Specifically, the ORs for an IQR increase in exposure were 1.06 (95% CI: 1.01–1.10) for PM_2.5_, 1.15 (95% CI: 1.08–1.21) for PM_10_, 1.07 (95% CI: 1.00–1.14) for NO_2_, 1.13 (95% CI: 1.07–1.19) for SO_2_, and 1.06 (95% CI: 1.01–1.11) for CO in the fully adjusted model.

**Table 4:** The odds ratios of hyperuricemia associated with an IQR increase according to 1-year exposure to air pollutants

Hyperuricemia Odds ratio (95% confidence interval)								
Air pollutant	Crude	*p*-value	Model 1^*^	*p*-value	Model 2^†^	*p*-value	Model 3^‡^	*p*-value
PM_2.5_	1.03 (0.99, 1.08)	0.109	1.03 (0.99, 1.07)	0.166	1.05 (1.01, 1.10)	0.017	1.06 (1.01, 1.10)	0.012
PM_10_	1.12 (1.06, 1.18)	< 0.001	1.11 (1.06, 1.17)	< 0.001	1.16 (1.10, 1.22)	< 0.001	1.15 (1.08, 1.21)	< 0.001
NO_2_	1.07 (1.00, 1.13)	0.040	1.05 (0.99, 1.12)	0.130	1.09 (1.03, 1.17)	0.005	1.07 (1.00, 1.14)	0.045
SO_2_	1.12 (1.07, 1.18)	< 0.001	1.12 (1.06, 1.17)	< 0.001	1.16 (1.10, 1.21)	< 0.001	1.13 (1.07, 1.19)	< 0.001
CO	1.05 (0.99, 1.10)	0.056	1.04 (0.99, 1.09)	0.146	1.07 (1.02, 1.12)	0.008	1.06 (1.01, 1.11)	0.022

^*^Adjusted for age and sex

^†^Adjusted for variables in model 1 and BMI, WC, smoking, alcohol consumption, and physical activity

^‡^Adjusted for variables in model 2 and eGFR, hypertension, diabetes mellitus, and dyslipidemia

*HU* hyperuricemia, *IQR* interquartile range, *PM* particulate matter, *BMI* body mass index, *WC* waist circumference, *eGFR* estimated glomerular filtration rate

### Subgroup analyses and effect modification

**Fig. 2:**
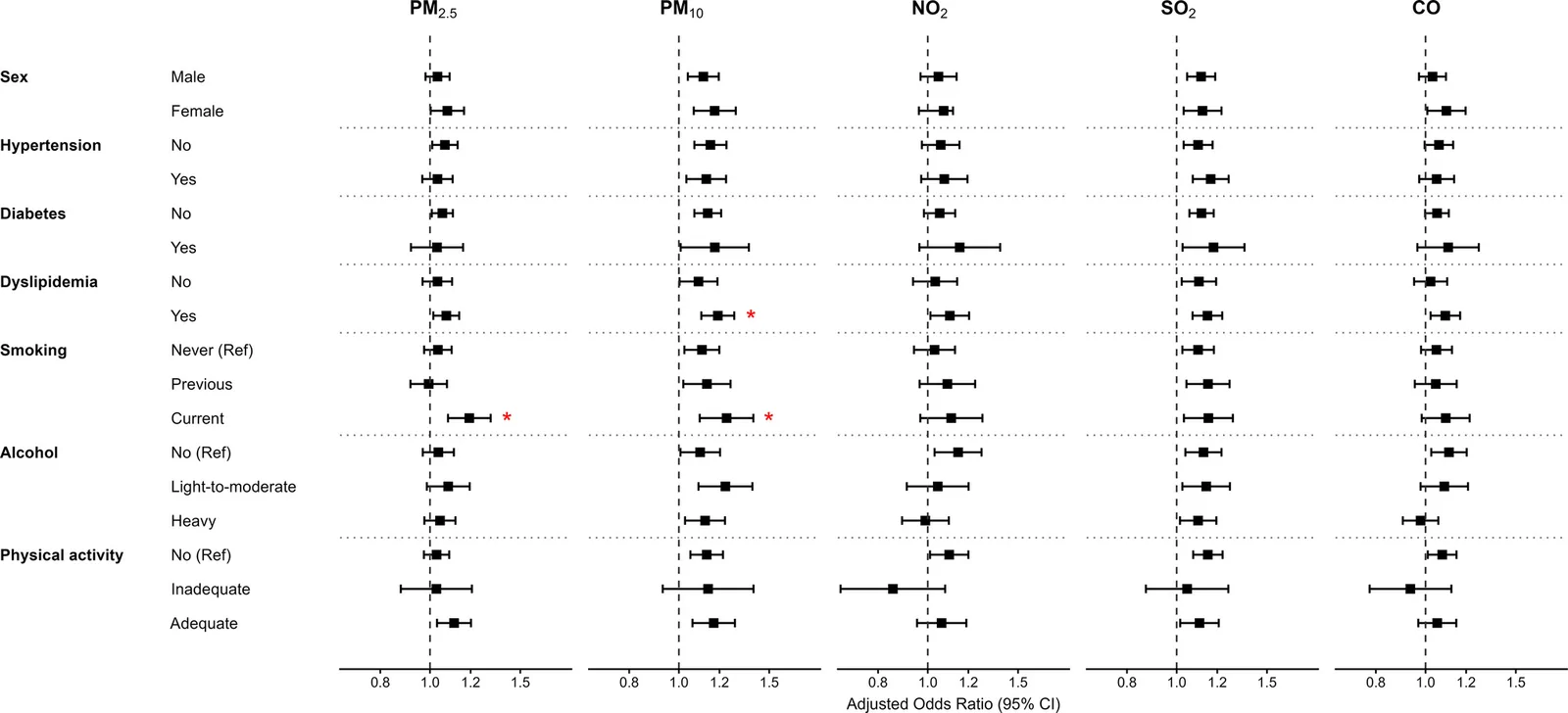
Stratified associations between 1-year average air pollutant concentrations and hyperuricemia by categorical covariates. * *p*-value < 0.05. Note: The squares represent the ORs, and the horizontal lines indicate the 95% CIs. The analyses were adjusted for age, sex, BMI, waist circumference, smoking status, alcohol consumption, physical activity, eGFR, and comorbidities (excluding the stratification variable in each subgroup). PM_2.5_, particulate matter 2.5; PM_10_, particulate matter 10; SO_2_, sulfur dioxide; NO_2_, nitrogen dioxide; CO, carbon monoxide; ORs, odds ratios; CIs, confidence intervals; eGFR, estimated glomerular filtration rate

Significant interactions were identified between air pollutants and several covariates in the multivariable logistic regression models. Among the categorical covariates, current smoking significantly modified the association between PM_2.5_ and hyperuricemia, with a stronger association observed in current smokers (Fig. 2). For PM_10_, significant interactions were observed among current smoking and dyslipidemia, with the odds of hyperuricemia notably higher among current smokers and participants with dyslipidemia than among their counterparts. Among the continuous covariates, age exhibited significant interactions with NO_2_ and SO_2_. The positive associations between these pollutants and hyperuricemia tended to increase with age. Additionally, a significant interaction was observed between WC and NO_2_, indicating that the odds ratio for hyperuricemia associated with NO_2_ exposure increased with higher WC (Supplementary Fig. 2).

## Discussion

This large-scale cross-sectional study demonstrated significant positive associations between long-term exposure to air pollutants (PM_2.5_, PM_10_, NO_2_, SO_2_, and CO) and elevated serum uric acid levels and hyperuricemia. These associations remained robust after adjustment for a comprehensive set of confounding factors, including lifestyle habits and metabolic comorbidities. The findings suggest that ambient air pollution is an independent environmental risk factor for hyperuricemia and contributes to variations in uric acid homeostasis.

The biological plausibility of these findings is supported by several potential nonrenal metabolic mechanisms, predominantly involving systemic inflammation and oxidative stress. Upon exposure to inhaled pollutants, airway epithelial cells overproduce mucosal uric acid through xanthine dehydrogenase upregulation [21]. While initially protective, excessive uric acid acts as a damage-associated molecular pattern that triggers local pulmonary inflammation [22]. This surplus uric acid is then rapidly absorbed into the systemic bloodstream via the highly vascularized alveolar network. This localized inflammation also induces the production of reactive oxygen species, causing oxidative damage to DNA and RNA. Beyond accelerated purine catabolism from the degradation of damaged nucleotides, air pollution directly perturbs purine metabolism within liver tissues [23]. Specifically, pollutant-induced systemic inflammation and oxidative stress amplify hepatic purine metabolism by promoting the immunometabolic upregulation of xanthine oxidoreductase—the key enzyme in uric acid synthesis—via proinflammatory cytokines, thereby accelerating uric acid overproduction [23, 24]. Furthermore, exposure to air pollutants induces hepatic and systemic insulin resistance, which overactivates the pentose phosphate pathway—an alternative intracellular energy route—ultimately accelerating de novo purine synthesis and elevating uric acid levels. In addition, this exposure increases the levels of precursor amino acids, such as glutamine and glycine, further contributing to uric acid accumulation. This indicates that air pollutants actively interfere with both amino acid and nucleic acid metabolism, thereby driving uric acid production [25, 26].

These metabolic disruptions and subsequent elevated uric acid levels have direct clinical implications for gout development. Recent epidemiological and experimental evidence highlights that both short-term acute spikes and long-term chronic exposure to ambient air pollutants are significantly associated with an increased incidence of gout flares [27, 28]. The mechanistic transition from asymptomatic hyperuricemia to acute gout flares involves the direct effects of air pollutants on the innate immune system [29]. Airborne particulate matter, upon phagocytosis by macrophages, acts synergistically with deposited monosodium urate crystals to strongly activate the NLRP3 inflammasome [29]. Activation of this inflammasome triggers the robust secretion of proinflammatory cytokines, such as interleukin-1β [29]. Therefore, air pollution exacerbates the risk of clinical gout not merely through the gradual elevation of serum uric acid but also by actively priming the macrophage NLRP3 inflammasome and amplifying the innate inflammatory response to urate crystals.

The subgroup analyses revealed that the adverse effects of air pollution were more pronounced among vulnerable populations. Specifically, current smokers exhibited a stronger association between PM exposure and hyperuricemia. Cigarette smoking impairs mucociliary clearance and induces chronic inflammation, which may act synergistically with air pollutants to amplify systemic oxidative stress [30]. Similarly, participants with dyslipidemia exhibited increased susceptibility to the hyperuricemic effects of PM_10_. Dyslipidemia is associated with endothelial dysfunction and a pro-inflammatory state. Exposure to air pollutants may further exacerbate lipid peroxidation and inflammatory responses [31]. Regarding continuous variables, the risk of hyperuricemia associated with NO_2_ and SO_2_ increased with age. This may be the result of immunosenescence and a decline in antioxidant defense mechanisms among older adults, making them more susceptible to environmental oxidative insults [32]. Additionally, the interaction between NO_2_ and waist circumference suggested that central obesity, characterized by chronic low-grade inflammation, exacerbates the metabolic disruption caused by air pollution [33].

A key strength of this study is the use of a representative sample of the general population, which distinguishes it from previous studies. Most previous longitudinal studies on air pollution and hyperuricemia included specific occupational groups or demographic segments, with limited generalizability. For example, Tang et al. focused exclusively on traffic police officers, a group with high occupational exposure to vehicle exhaust fumes, and their sample was predominantly composed of males [12, 13]. Conversely, Duan et al.’s study, which was conducted among government employees, included a predominantly female sample, thereby limiting sex balance [10]. Similarly, Hu et al. analyzed data from the CHARLS cohort, which predominantly included middle-aged and older adults, limiting the applicability of their findings to younger populations [11]. Conversely, the present study included participants from a general health-screening cohort with a wide range of age groups, a balanced sex ratio, and diverse occupational backgrounds. This minimized selection biases related to specific occupations, sex ratio, and age-related physiological conditions, thereby enhancing the findings’ generalizability to the broader public.

Another distinctive feature of this study was the demonstration of adverse health effects at relatively low concentrations of specific air pollutants compared with those reported in previous studies conducted in highly polluted regions, such as China [10–13]. For example, the mean annual concentration of PM_2.5_ in this study was 24.55 μg/m^3^, which is considerably lower than the levels reported in the aforementioned Chinese cohorts (39.35–57.92 μg/m^3^). However, this study observed that even a small increment of 1 IQR (2.62 μg/m^3^) in air pollutant exposure was significantly associated with increased uric acid levels. This finding supports the hypothesis that there is no safe threshold for air pollution exposure and emphasizes that even moderate to low levels of air pollution can pose a significant public health burden from metabolic diseases [34].

Furthermore, this study provides crucial insights into the mechanisms linking air pollution to hyperuricemia by demonstrating that this association is independent of renal function. Previous studies established that air pollution is a risk factor for CKD and reduced eGFR, both of which can secondarily lead to uric acid retention [35, 36]. However, although this study adjusted for eGFR in the regression models, the association between air pollutants and hyperuricemia remained statistically significant. This suggests that elevated uric acid levels are not solely a consequence of impaired renal excretion. Instead, air pollution could directly stimulate the overproduction of uric acid via oxidative stress and metabolic disruption, independent of its nephrotoxic effects [6–9].

This study had several limitations. First, exposure estimations based solely on residential addresses may not have fully captured individual exposure variations arising from daily mobility or indoor air quality. Future studies should incorporate detailed information on daily mobility patterns and specific locations where participants spend significant time (e.g., workplaces or schools). By accounting for these time-weighted locations rather than relying exclusively on static residential addresses, researchers can achieve a more precise and dynamic estimation of personal exposure. Second, despite adjustments for individual covariates, unmeasured socioeconomic factors influencing residential choices may have introduced residual confounding regarding regional pollution levels. To mitigate this, subsequent research should integrate comprehensive socioeconomic indicators, such as area-level deprivation indices or household income data. Third, this study lacked detailed data on dietary intake (e.g., purine-rich foods), which is a direct determinant of serum uric acid levels. Ideally, future investigations should employ nutritional logs to rigorously control for dietary confounders. Fourth, our analysis did not adjust for severe clinical outcomes, such as a history of cardiovascular events or neurodegenerative disorders (e.g., Alzheimer's disease). Specific diagnostic data for neurodegenerative disorders were unavailable in our general health screening cohort. Additionally, we could not adequately evaluate the history of cardiovascular events due to data limitations, including a high non-response rate on self-reported questionnaires, potential inaccuracies in self-reported diagnoses, and the relatively low prevalence of these severe conditions among an asymptomatic population undergoing routine health screenings. Therefore, we were unable to explore the relationship between hyperuricemia, air pollution, and these severe neurological and cardiovascular outcomes. Finally, owing to the cross-sectional design, the temporal sequence could not be determined, limiting this study’s ability to infer a causal relationship between air pollution and hyperuricemia. Large-scale prospective cohort studies with repeated measurements of both air pollution and uric acid levels are needed to elucidate the causal nature of this association.

In conclusion, this study provides robust evidence that long-term exposure to ambient air pollutants, including PM_2.5_, PM_10_, NO_2_, SO_2_, and CO, is significantly associated with an increased risk of hyperuricemia. Notably, these associations persisted independently of renal function, suggesting that air pollution may directly disrupt uric acid homeostasis, potentially through oxidative stress and systemic inflammation pathways. Furthermore, significant associations were observed even at relatively low concentrations of air pollutants, with stronger associations found among vulnerable subgroups, such as smokers, older adults, and individuals with dyslipidemia. These findings emphasize the lack of a safe threshold of air pollution exposure and its implications for metabolic health. Therefore, stricter environmental regulations and targeted public health strategies are essential to mitigate the burden of air pollution-related metabolic diseases, including hyperuricemia and cardiovascular diseases, among the general population.

## Supplementary Information

Below is the link to the electronic supplementary material.

ESM 1 Pearson correlation coefficients among air pollutants. Note: Exposure concentrations were calculated as the average of the daily concentrations during the 365 days leading up to each participant's screening date. PM2.5, particulate matter 2.5; PM10, particulate matter 10; SO2, sulfur dioxide; NO2, nitrogen dioxide; CO, carbon monoxide (PNG 111 KB)

Supplementary file1 (TIF 1130 KB)

ESM 2 Marginal effects plots illustrating the interactions between air pollutants and continuous covariates. (A) Interaction between NO2 and age; (B) Interaction between NO2 and waist circumference; (C) Interaction between SO2 and age Note: The solid lines represent the estimated odds ratios of hyperuricemia, and the shaded areas indicate the 95% confidence intervals (PNG 249 KB)

Supplementary file2 (TIF 1365 KB)

## Data Availability

The datasets generated and/or analyzed during the current study are not publicly available due to privacy and ethical restrictions regarding individual health information. However, they are available from the corresponding author (Jin Ho Park) upon reasonable request, subject to approval by the Institutional Review Board of Seoul National University Hospital.
